# Conformation of methylated GGQ in the Peptidyl Transferase Center during Translation Termination

**DOI:** 10.1038/s41598-018-20107-8

**Published:** 2018-02-05

**Authors:** Fuxing Zeng, Hong Jin

**Affiliations:** 1 Department of Biochemistry, University of Illinois at Urbana-Champaign, Urbana, USA; 2 Center for Biophysics and Quantitative Biology, University of Illinois at Urbana-Champaign, Urbana, USA

## Abstract

The universally conserved Gly-Gly-Gln (GGQ) tripeptide in release factors or release factor-like surveillance proteins is required to catalyze the release of nascent peptide in the ribosome. The glutamine of the GGQ is methylated post-translationally at the N^5^ position *in vivo*; this covalent modification is essential for optimal cell growth and efficient translation termination. However, the precise conformation of the methylated-GGQ tripeptide in the ribosome remains unknown. Using cryoEM and X-ray crystallography, we report the conformation of methylated-GGQ in the peptidyl transferase center of the ribosome during canonical translational termination and co-translation quality control. It has been suggested that the GGQ motif arose independently through convergent evolution among otherwise unrelated proteins that catalyze peptide release. The requirement for this tripeptide in the highly conserved peptidyl transferase center suggests that the conformation reported here is likely shared during termination of protein synthesis in all domains of life.

## Introduction

Appropriate termination of protein synthesis is essential to achieve translational accuracy and fidelity. Translation normally terminates at the end of a coding sequence when a stop codon is encountered. In canonical translation termination, a protein release factor binds to the aminoacyl site (A-site) of the ribosome and catalyzes the release of the nascent peptide in the ribosomal peptidyl site (P-site)^1–3^. In a similar manner, a release factor-like surveillance protein is often recruited by the ribosome to terminate protein synthesis in the co-translational quality control process to prevent formation of abnormal protein products when errors occur^4,5^. In these proteins, the GGQ motif required for the catalytic activity of peptide release is universally conserved^6–8^. The conformation revealed by structures of canonical termination complexes shows that the backbone of the two Gs assumes torsion angles only accessible to glycine^9–13^. This satisfactorily explains why any mutation in both glycines completely abolishes peptide release activity. By contrast, the conformation of the glutamine in the GGQ motif remains obscure. However, biochemical^14,15^, structural^9^ and computational^16,17^ investigations have demonstrated unambiguously that the sidechain of the glutamine assumes a packing role in the peptidyl transferase center (PTC) for releasing the nascent peptide.

**Figure 1:**
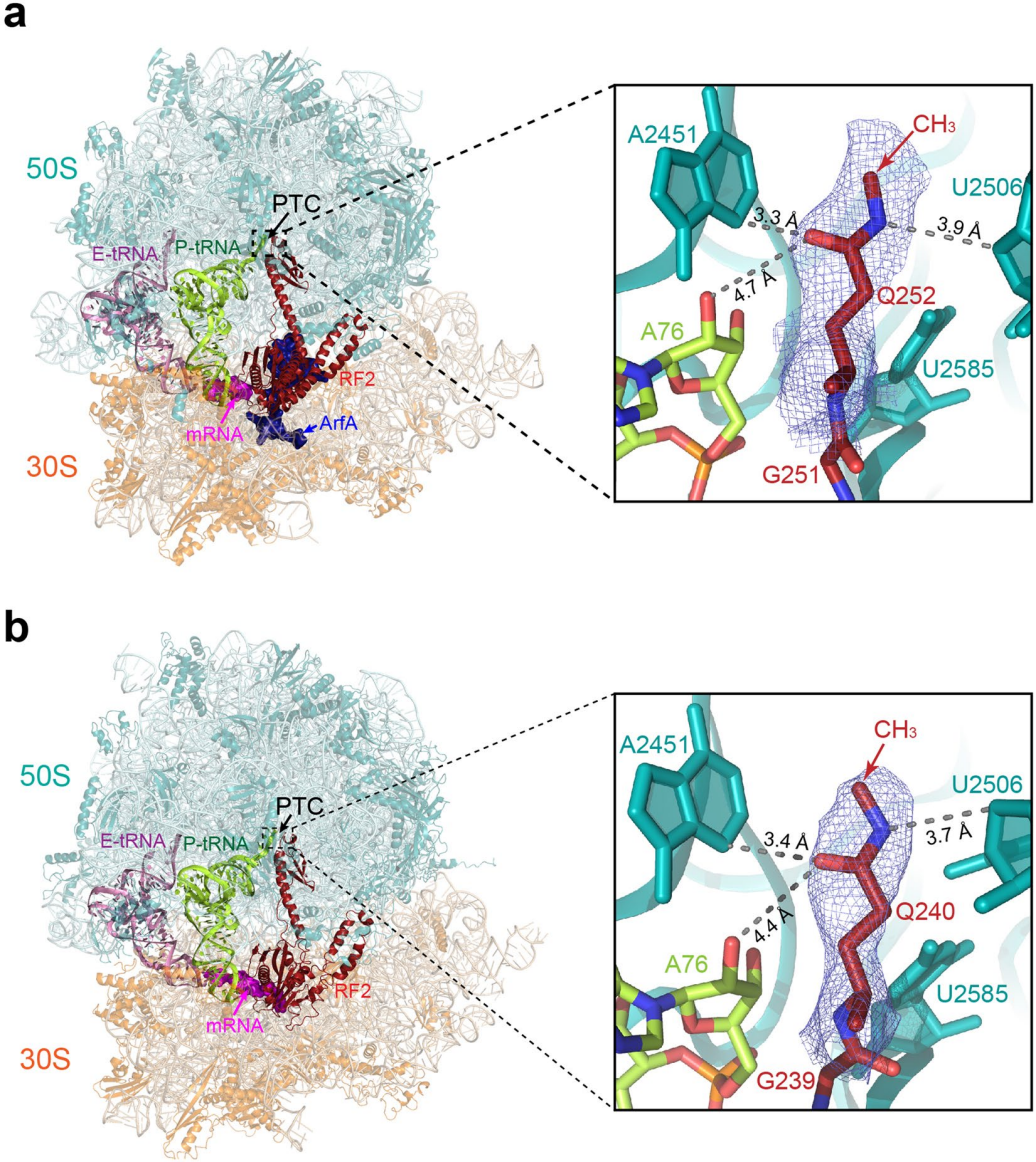
Conformation of methylated GGQ in the PTC during translation termination. **(a)** CryoEM structure of a nonstop termination complex of the *E. coli* 70S ribosome with tRNA^fMet^ in the E- (pink) and P-site (lemon), a nonstop mRNA (magenta), ArfA (dark blue) and RF2 (dark red) in the A-site. Zoomed view (right panel) shows the cryoEM map at σ = 2.0 contour level for the glutamine (Q252) of the GGQ motif in the PTC. The map of the glutamine sidechain shows the orientation of the carbonyl and N^5^-methyl groups. The distance between the N^5^H amine to the O4′ of U2506 is 3.9 Å. The carbonyl oxygen of the glutamine sidechain is separated from the N^3^ of A2451 and the 2′OH of A76 by 3.3 Å and 4.7 Å, respectively. 23S rRNA, RF2 and P-tRNA are coloured in teal, dark red and lemon. The coordinates for the overall view are from global refinement of the nonstop termination complex, and the coordinates for the zoomed view are from local refinement over the PTC region. **(b)** The crystal structure of the *T. thermophilus* 70S ribosome in complex with E-tRNA (pink), P-tRNA (lemon), mRNA with stop codon UGA (magenta) and *T. thermophilus* RF2 (dark red). Zoomed view (right panel) shows a *2mF*_*obs*_*-DF*_*cal*_ map (σ = 1.0) of the PTC. The methylated GGQ motif in the canonical termination complex adopts the same conformation as seen in the nonstop termination complex (**a**). Important distances between atoms of the glutamine and surrounding residues of the PTC are shown.

Notably, the glutamine in the GGQ motif is invariably methylated post-translationally at the *N*^5^ position^18,19^. This covalent modification has been reported to enhance peptide release, particularly in the RF2-dependent pathway in *E. coli in vitro*^15,18,20^. The same modification in *S. cerevisiae* is required for optimal cell growth^21^. *In vitro* kinetic experiments demonstrated a significant entropic and enthalpic contribution conferred by the methylation to the activation energy of the release reaction^15^. Consistent with an enhanced activity, a recent study showed that methylation of GGQ increases the rate of peptide release on glycine- and proline-containing peptides, which are otherwise slow in *in vitro* reactions^22^. In-line with the biochemical results, computational studies also showed that *N*^5^-methylation enhances the packing of the glutamine in the catalytic site^17^. Therefore, the precise conformation of the glutamine in the PTC must be influenced by methylation; however, this conformation remains unknown during translation termination. Here, we report atomic resolution structures of both a canonical and a quality control termination complex that reveal the conformation of methylated GGQ in the PTC of the ribosome. The structure of the canonical termination complex was solved by X-ray crystallography at 3.2 Å, and the quality control termination complex was determined by single-particle electron cryo-microscopy (cryoEM) with a resolution of 3.1 Å **(**Fig. 1**;** Supplementary Figs S1, S2 and S3**;** Supplementary Tables S1 and S2**)**.

## Results and Discussion

**Figure 2:**
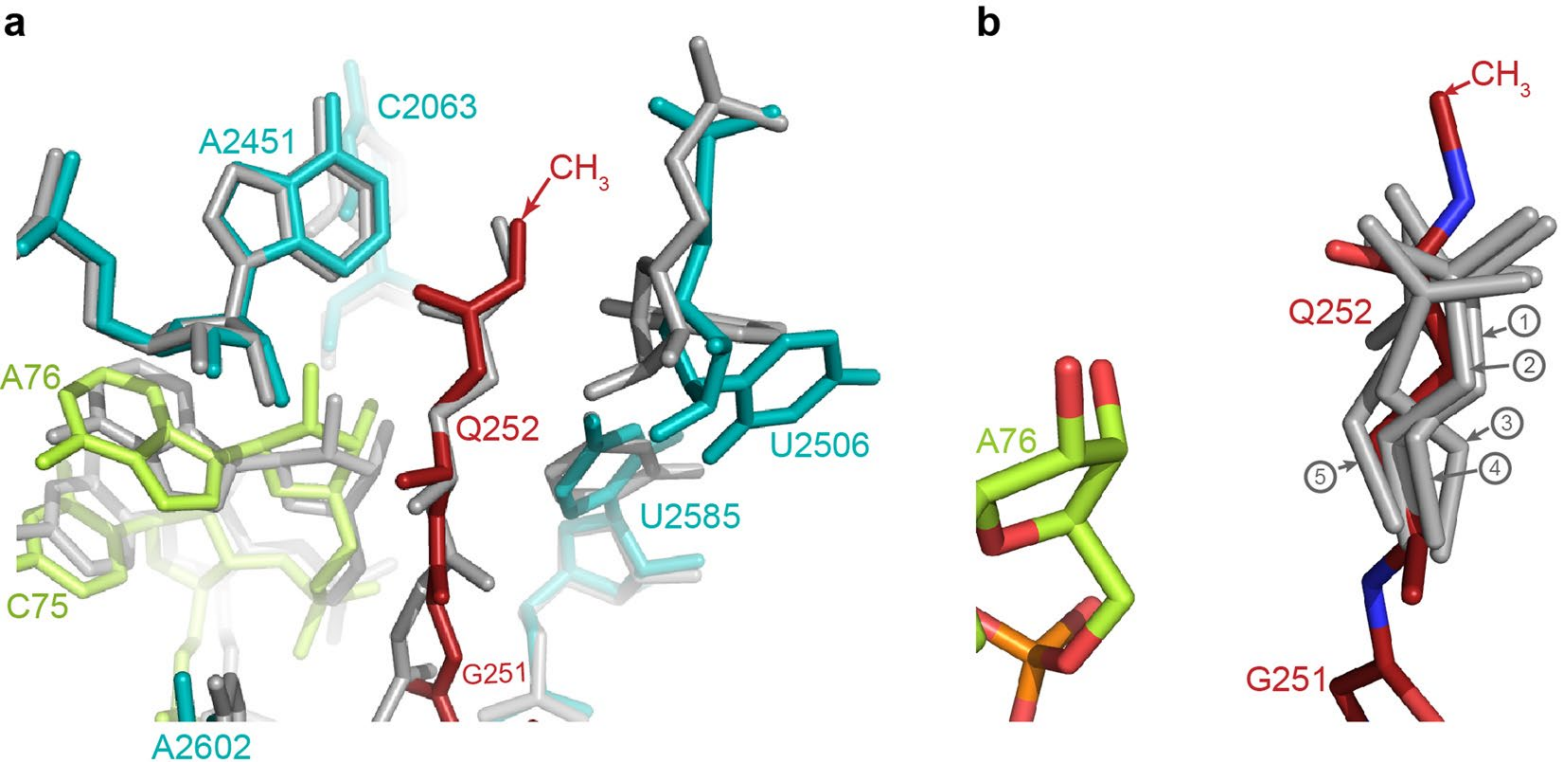
Conformations of methylated and unmethylated GGQ motifs in the PTC during translation termination. (**a**) Conformations of the methylated GGQ motif in the PTC. Superposition of the 23S rRNAs from structures of the canonical termination complex from *T. thermophilus* (grey) and the nonstop translation complex from *E. coli* (coloured in red for RF2^m^, cyan for 23S, and lemon for P-tRNA) showing similar conformations of the glutamine in the GGQ motif. In both structures, the carbonyl oxygen of the glutamine side chain orients toward the base of A2451 and P-tRNA. It’s remains to be understood whether the different conformations of U2506 between the two structures have mechanistic implications regarding the function of this residue in canonical versus nonstop termination. Backbone differences of G250 and G251 in the GGQ motif may be due to sequence variation between RF2 in *E. coli* and *T. Thermophilus*. In *E. coli* RF2, an alanine preceeds the GGQ, whereas the preceeding residue is proline in *T. thermophilus* RF2. **(b)** Side chains of the unmethylated glutamine in the GGQ motif determined by previous structural investigations assume different conformations in the PTC. Unmethylated glutamine displays conformational diversity (grey) compared to the structures determined in this study (dark red); *N*^5^-methylation of the glutamine stabilizes the side chain orientation. P-tRNA is shown as sticks in lemon. The 23S of the ribosome was used for alignment. The 70S termination complexes with RF2 and stop codon UAA (①, PDB: 4V67)^10^; with RF1 and stop codon UAG (②, PDB: 4V7P)^11^; with RF1 on stop codon UAA (③, PDB: 4V63)^12^; with RF2 on stop codon UGA (④, PDB: 4V5E)^13^; with RF2 on stop codon UAA (⑤, PDB: 4V5J)^9^.

The *E. coli* 70S ribosome complexed with *E. coli* ArfA and RF2 on a truncated mRNA was used as a representative for the ribosomal complex involved in the co-translational quality control pathway. ArfA/RF2-dependent nonstop recognition is one of the three known pathways in bacteria for rescuing ribosomes that are translating mRNAs without a stop codon^4^. ArfA detects the presence of a truncated mRNA on the ribosome by inserting its positively charged C-terminal tail into the empty mRNA channel. Once bound to the ribosome, ArfA recruits RF2 and induces RF2 to its catalytically competent “open” conformation for releasing the nascent peptide^23–27^. The *T. thermophilus* ribosome complexed with *T. thermophilus* release factor RF2 and mRNA containing a UGA stop codon was used as a canonical termination complex. In both canonical and nonstop termination complexes, the orientation of the methylated glutamine sidechain assumes the same well-defined conformation (Fig. 2a**)**, which differs significantly from previously determined conformations of unmodified GGQ motifs (Fig. 2b). The post-translationally added CH_3_ group orients towards the nascent peptide exit tunnel, positioning its -N^5^H- imino group close to the O4′ of the ribose in U2506. The carbonyl oxygen of the glutamine sidechain points towards the base of A2451 and A76 of the P-tRNA. The distances between the carbonyl oxygen of the glutamine to N^3^ of A2451 and to the 2′OH of A76 are 3.3 Å and 4.7 Å, respectively (Fig. 1a).

**Figure 3:**
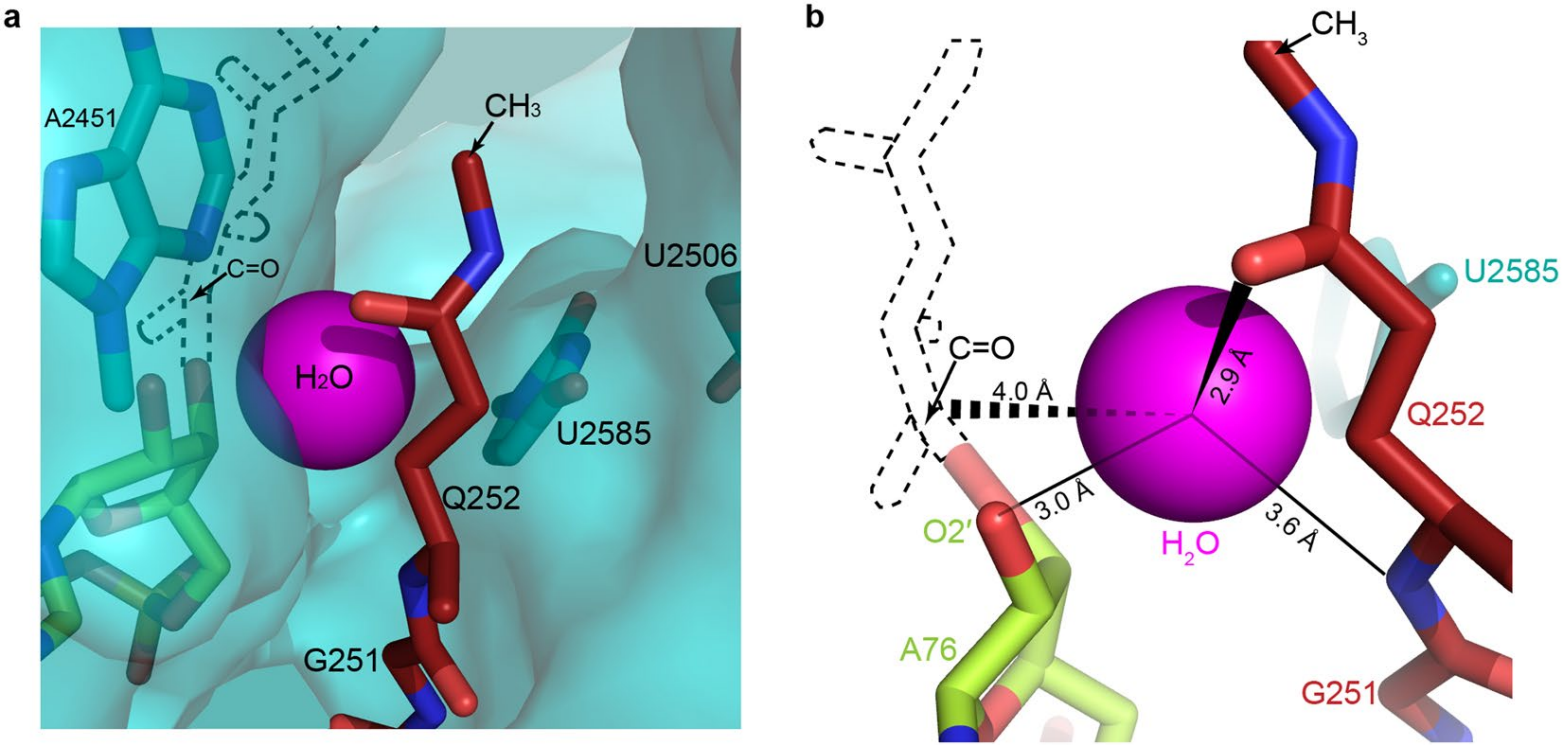
The catalytic pocket of the PTC in translation termination. Catalytic pocket in the PTC showing one water molecule (magenta) can be accommodated (**a**) and the catalytic water can be coordinated by the main chain NH and side chain carbonyl oxygen of Q252 and 2′ OH of A76 (**b**). In this way, a lone pair of electrons from the water can nucleophilically attack the carbonyl carbon in the ester bond betwee the P-site tRNA and nascent peptide for the hydrolysis reaction. Nascent peptide (dashed lines) connected with A76 was modeled according to PDB: 3J92^58^.

Compared to various structures of unmodified glutamine reported previously (Fig. 2b and Supplementary Fig. S4), the *N*^5^-methylation stabilizes the glutamine conformation through increased van der Waals interactions with three important residues in the PTC: U2506, A2451 and A2452. Chemically, peptide release is achieved by hydrolysis of the ester bond between the nascent peptide and peptidyl-tRNA (P-tRNA) in the ribosome, and earlier kinetic data suggest that the water molecule required for this process needs to be activated for catalysis to take place^14,28^. As shown in Fig. 3a, the catalytic pocket created by polar groups, including N^3^ in the base of A2451, 2′OH of A76, and the mainchain amide and sidechain carbonyl oxygen of the glutamine, is just right to accommodate one water molecule in the RNA-rich core of the PTC for the hydrolysis reaction. Based on the structure, one modeled water can be coordinated by hydrogen bonding with the 2′OH of A76, the sidechain carbonyl oxygen and mainchain amide of the glutamine, and in this way one lone pair of electrons remains available for nucelophilic attack on the carbonyl carbon in the ester bond at the P-site (Fig. 3b).

Consistent with our structural observations, *N*^5^-methylation of the glutamine promotes peptide release on stop codons during canonical translation termination (Table 1 and Supplementary Fig. S5), as well as on nonstop mRNAs by ArfA/RF2^20^. Methylation of the GGQ sequence marginally affects the *K*_*1/2*_ for binding of the release factors to their cognate codons (Table 1). However, the same covalent modification enhances the *k*_*cat*_ of peptide release for both RF1 and RF2. The rate enhancement for RF1 is modest (~5-fold), whereas the enhancement for RF2 is ~20-fold. Finally, comparison of the kinetics between the cognate and near-cognate complexes suggests the coupling between correct binding of a stop codon and catalysis is less tight for RF2 compared to RF1 (Table 1 and Supplementary Fig. S5), consistent with earlier biochemical work^29^. Notably, the orientation of the glutamine sidechain in our experimentally determined structure agrees well with earlier theoretical structural models^11,17,30^, but does not support an alternative model suggested from a recent crystal structure of the heterologous canonical termination complex of *T. thermophilus* ribosome and *E. coli* RF1^22^. In the structure of this heterologous canonical termination complex, the post-translationally added methyl group was reported to point to the base of A2451. Instead of the carbonyl oxygen, the -N^5^H- was modeled to face A76 of the P-tRNA, although the electron density to support the model fitting was less convincing. Furthermore, in our structure, an ~180-degree rotation around the C_δ_-C_γ_ bond in the glutamine swaps the positions of carbonyl and methyl-NH groups, but the methyl group resides outside of the density. A refinement of this rotamer with Refmac or Phenix showed that the overall fitting of this alternative glutamine conformation to the density is not as good as our structure shown in Fig. 1 (Supplementary Fig. S6). Lastly, it’s worth mentioning that sequences of bacterial release factors from different species vary, yet these proteins resemble each other structurally. Therefore, it remains questionable whether the conformation from a heterologous termination complex is functionally pertinent, as suggested by a decreased rate constant for peptide release compared to the homologous complex (Jin Lab unpublished data), and also seen from one heterologous termination complex recently^25^. In this study, the *E. coli* ArfA can recruit *T. Thermophilus* RF2 but fails to induce the RF2 into the “open” or catalytically competent conformation in the *T. Thermophilus* ribosome.

**Table 1:** Kinetic parameters of peptide release by methylated and unmethylated release factors on cognate and near-cognate stop codons in the *E. coli* ribosome *in vitro*.

Release Complexes	A site codon	*k*_*cat*_ (s^-1^)	*K*_*1/2*_ (10^−6^ M)	*k*_*cat*_ */K*_*1/2*_	*k*_*cat*_ (s^−1^)	*K*_*1/2*_ (10^−6^ M)	*k*_*cat*_ */K*_*1/2*_
		RF2^m^			RF2		
Cognate Complexes	UGA*	0.083 ± 0.001	0.07 ± 0.01	1.2	0.0091 ± 0.0002	0.11 ± 0.01	0.08
	UAA	0.108 ± 0.001	0.21 ± 0.03	0.5	0.0052 ± 0.0003	0.08 ± 0.03	0.07
Near-cognate complexes	UGG*	0.0039 ± 0.0006	4.10 ± 1.46	0.0009	0.0010 ± 0.0001	4.58 ± 0.76	0.0002
		**RF1** ^**m**^			**RF1**		
Cognate complexes	UAG	1.43 ± 0.03	0.07 ± 0.02	20	0.309 ± 0.008	0.08 ± 0.01	3.9
	UAA	1.24 ± 0.01	0.14 ± 0.01	8.8	0.289 ± 0.007	0.09 ± 0.01	3.2
Near-cognate complexes	UGG	0.0069 ± 0.0010	3.30 ± 0.70	0.002	0.0027 ± 0.0002	3.20 ± 0.40	0.0008

*Data were taken from the previous study^20^ for the purpose of comparison. Catalytic rate constants *k*_*cat*_ and values of *K*_*1/2*_ were obtained by fitting the observed rates against the corresponding release factor concentrations to the Michaelis–Menten equation. An average of three independent measurements is reported for each reaction and errors are calculated by standard error propagation.

Taken together, the post-translational modification helps to stabilize the glutamine conformation in the PTC of the ribosome, and orients the carbonyl oxygen of the glutamine to coordinate a water nucleophile in the hydrolysis reaction. Therefore, precise structural orientation of the glutamine sidechain conferred by N^5^ methylation allows for better accommodation of the water molecule during peptide release. The GGQ tripeptide has evolved to catalyze the hydrolysis reaction in the PTC of the ribosome. The highly conserved nature of this peptide motif and the ribosomal PTC suggest the conformation of the GGQ reported in this study will most likely be adopted by eukaryotic class I release factor eRF1 and other release factor-like surveillance proteins in higher organisms.

## Methods

### Purification of ribosomes, tRNAs and proteins

*Escherichia coli* ribosomes from MRE600 strain and *Thermus thermophilus* ribosomes from K8 strain were purified as described^13,31^. *E. coli* tRNA^fMet^ and tRNA^Phe^ were prepared by *in vitro* transcription^9,20^. Release factors from *E. coli* and *T. thermophilus* were cloned and overexpressed in BL21 (DE3) cells and purified by affinity and ion-exchange chromatography^9,20^. *E. coli* and *T. thermophilus* release factors with GGQ motif methylated (RF1^m^ and RF2^m^) were obtained by co-expressing the release factors and their cognate methyltransferase (PrmC) gene together and the methylation status of these proteins was identified by mass spectroscopy as described^20,27^.

mRNAs with AUG or UUC in the P-site and different cognate or near-cognate stop codons in the A-site were purchased from Dharmacon (GE, Thermo Scientific). Sequences of the mRNAs are listed as the following with the P-site codon underlined:

SD-PMet-UAA: 5′-GGC AAG GAG GAA AAA AUG UAA UAC A-3′

SD-PMet-UGA: 5′-GGC AAG GAG GAA AAA AUG UGA UAC A-3′

SD-PMet-UAG: 5′-GGC AAG GAG GAA AAA AUG UAG UAC A-3′

SD-PMet-UGG: 5′-GGC AAG GAG GAA AAA AUG UGG UAC A-3′

SD-PPhe-UGA: 5′-GGC AAG GAG GAA AAA UUC UGA UAC A-3′

### Peptide release assay

Aminoacylation and formylation of tRNA^fMet^ were done as described^20^. Translation termination complexes containing *E. coli* 70S ribosomes, f-[^35^S]-Met-tRNA^fMet^ and mRNAs with different cognate or near-cognate stop codons were programmed and the catalytic rate constants *k*_*cat*_ and binding constants *K*_*1/2*_ were measured as described^3,20,32^.

### Electron microscopy, data collection and image processing

Ribosomal complexes were formed by incubating ribosomes with mRNA (6 μM), tRNA^fMet^, ArfA and RF2^m^ (12 μM each) in buffer A (20 mM Hepes-KOH, pH 7.5, 15 mM magnesium acetate, 150 mM potassium acetate, 4 mM β-mercapthoethanol, 2 mM spermidine, 0.05 mM spermin) for 30 min at 37 °C^33^. Sample preparation for cryoEM followed the methods as described^34,35^. Data was collected in vitreous ice using a JEOL 3200FS transmission electron microscope operating at 300 keV equipped with a K2 summit direct electron detector (Gatan)^27^. Micrographs from the same nonstop complex^27^ were combined to achieve the best possible data quality. 2,107 micrographs showing Thon rings beyond 3.5 Å were used. The drifts of movie frames were corrected using MotionCor2^36^. Magnification anisotropy was corrected with mag_distortion_estimate and mag_distortion_correct^37^. CTFFIND4^38^ was used to determine the contrast transfer function.

Subsequently, a total of 268,364 particles were extracted and subjected to reference-free 2D classification in Relion^39,40^ to remove non-ribosomal particles. 3D classification was carried out over 221,911 particles to remove ribosomes without ligands bound (Supplementary Fig. S1). A total of 177,529 particles yielded from the 3D classification were refined using the cryoEM map of the same nonstop complex (EMD-8505)^27^ as an initial reference. Statistical particle-based movie correction and radiation-damage weighing^41^ were then used to obtain the polished particles. Finally, a focused classification with signal subtraction (FCwSS)^42^ using a soft-edged mask over P-tRNA and RF2 was performed. After additional rounds of 2D and 3D classifications, a total of 143,372 particles were subjected to refinement and yielded a global 3D reconstruction with an overall resolution of 3.24 Å.

A masked 3D refinement with partial signal subtraction was performed^42,43^ to obtain a better resolved map for the PTC region of the ribosome. A mask over the PTC was applied, and the final refinement led to a reconstruction with an overall resolution of 3.10 Å.

Resolutions were reported base on the gold-standard Fourier shell correlation (FSC) of 0.143 criterion^44,45^. Final map was sharpened by applying a negative B-factor estimated using Relion^40,44^. Local resolution was estimated using ResMap^46^.

### Model building and refinement

Coordinates for the same nonstop complex obtained from an earlier study (PDB: 5U4I)^27^ were used as starting models for the model building and refinement. Models were fitted into the maps using Chimera^47^ and built according to the map using Coot^48^.

Refinement was carried out in Refmac5.8^49^ with an optimized B-factor of −50. Molecular restraints for the secondary structure, RNA sugar pucker, base stacking and base-pair geometry were generated by ProSMART^50^ and LIBG^51^. Cross-validation against overfitting was done on two half maps where the FSC was monitored^51^, and the final model was validated using MolProbity^52^. Refinement statistics were summarized in Supplementary Table S1.

### Canonical translation termination complex formation and crystallization

Canonical termination complexes of the 70S ribosome and RF2^m^ from *T. thermophilus* on mRNA SD-PPhe-UGA with a stop codon UGA were formed in buffer B (5 mM Hepes pH 7.5, 10 mM Mg(OAc)_2_, 50 mM KCl, 10 mM NH_4_Cl and 6 mM β–mercaptoethanol). Crystallization of the complex was carried out as described^9,13^. Briefly, 2.8 mM Deocy Big Chap (Hampton Research) was mixed with the formed termination complex immediately before the crystallization. Crystals were grown at 19 °C in 100 mM Tris-HAc pH 7.12, 200 mM KSCN, 3.9–4.2% (w/v) PEG20K, 3.9–4.2% (w/v) PEG550MME and were cryoprotected by using 4.6% PEG20K and PEG550MME (4.6%, 10.95%, 17.3%, 23.65% and 30%). Crystals were harvested and plunge frozen in liquid nitrogen.

### Data collection, model building and refinement

X-ray diffraction data were collected at 100 K at the 24-IDC beamline at Northeastern Collaborative Access Team (NE-CAT) in the Advanced Photon Source, Argonne National Laboratory. Data were processed using XDS^53^. The apo *T. thermophilus* 70S ribosome from PDB code 4V51^54^ was used as the starting model. Refinement was carried out using CNS^55^ and Phenix^56^. The ligands including mRNA, E-tRNA and partial P-tRNA without the CCA-end, and partial RF2 without the GGQ motif were first built into unbiased difference density. After the refinement, the sigma-A weighted difference Fourier maps clearly showed the orientation of the methylated glutamine. The CCA end of the P-tRNA and the GGQ^m^ motif were built lastly. Refinement statistics were summarized in Supplementary Table S2.

Figures were generated using Chimera^47^ and PyMol^57^.

### Data availability

CryoEM maps for the 70S nonstop ribosomal complex and the PTC region have been deposited in the Electron Microscopy Data Bank under accession codes EMD-7341 and EMD-7340, respectively. Coordinates generated from cryoEM data have been deposited in the Protein Data Bank under accession code 6C4I for the 70S ribosome and 6C4H for the PTC region. The coordinate and structure factor files of the crystal structure on the canonical termination complex are deposited in the Protein Data Bank under accession code 6C5L.

## Electronic supplementary material

Supplementary figures
