# Supplementary material for: Conformation of methylated GGQ in the Peptidyl Transferase Center during Translation Termination
Source: Sci Rep. 2018 Feb 5;8:2349. doi: 10.1038/s41598-018-20107-8 (PMC5799190; doi:10.1038/s41598-018-20107-8)
Supplement: Supplementary file 1 — Supplementary figures [file 41598_2018_20107_MOESM1_ESM.pdf]

Conformation of methylated GGQ in the Peptidyl Transferase Center during Translation Termination

Authors: Fuxing Zeng[1] and Hong Jin[1,2,]*

Affiliations: [1.] Department of Biochemistry, [2.]Center for Biophysics and Quantitative Biology, University of Illinois at Urbana-Champaign

* Corresponding author

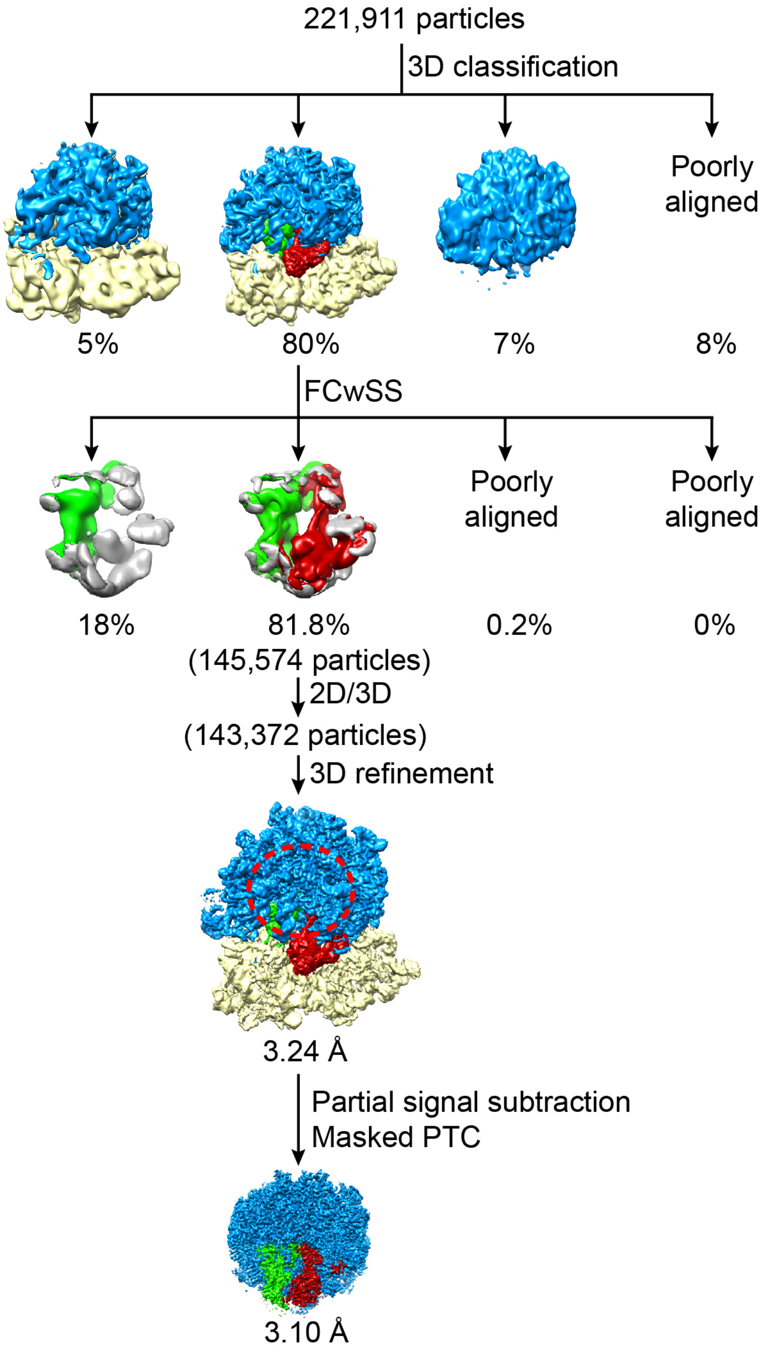

Supplementary Fig. S1 | Structure determination of the methylated RF2 in the PTC by cryoEM. Two rounds of 3D classification including an FCwSS step with the mask over P-tRNA and RF2 were performed on the selected 221,911 particles from 2D classification. The resulting 143,372 particles were refined to 3.24 Å. Finally, using a mask over the PTC, a local refinement with partial signal subtraction was carried out to improve the map quality. The final reconstruction over the PTC region has achieved a nominal resolution of 3.10 Å. 50S (blue), 30S (yellow), P-tRNA (lemon) and RF2 (firebrick) are shown.

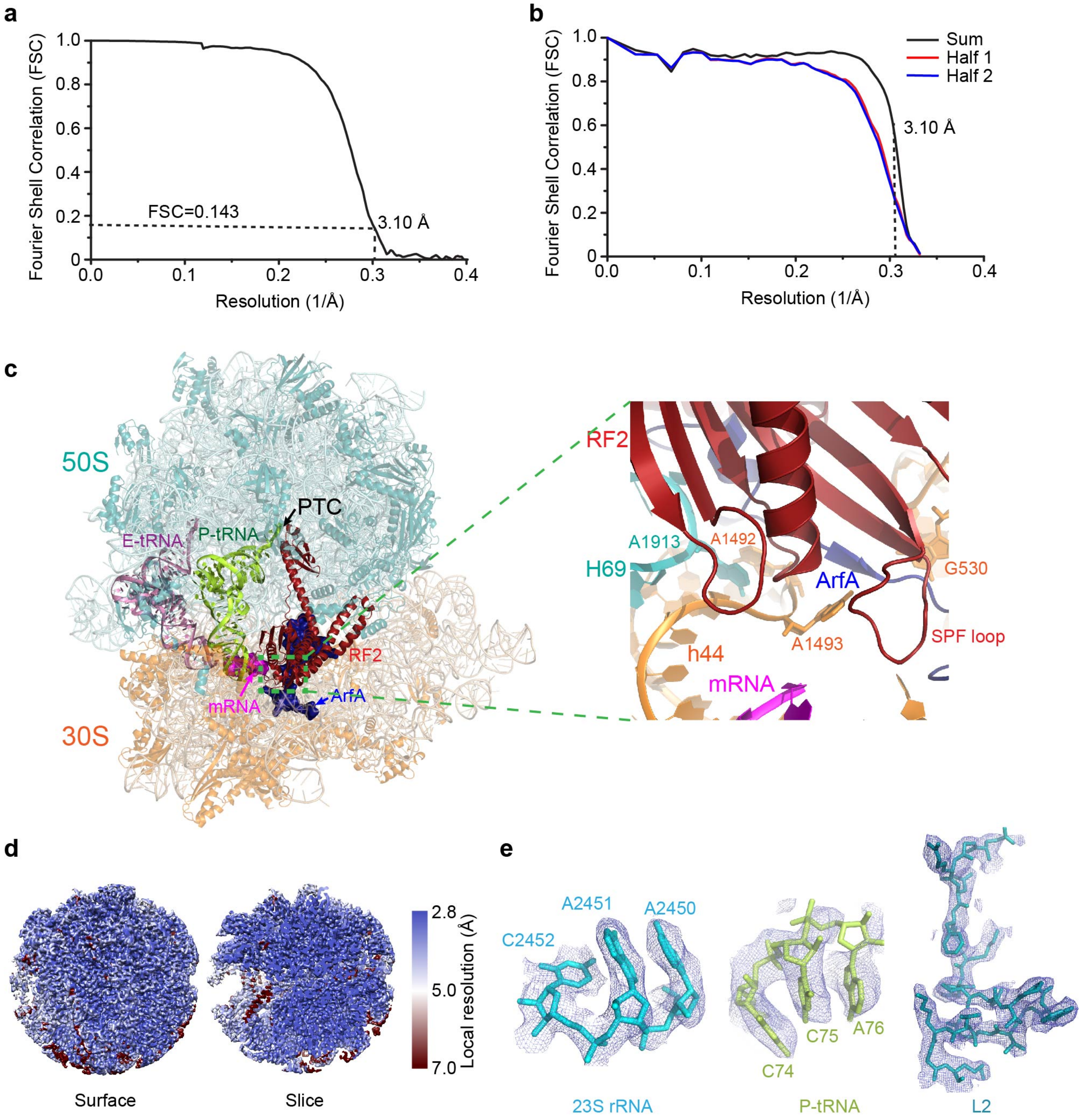

Supplementary Fig. S2 | Quality of map and structure determined by cryoEM.

(a) Gold-standard Fourier shell correlation (FSC) curves for the reported cryoEM reconstruction.

(b) Model cross-validation. FSC curves calculated between the refined structural model and cryoEM map (sum, black), with the self-validated (half1, red) and cross-validated (half2, blue) correlations shown.

(c) The structure of the *E. coli* 70S ribosome in complex with $tRNA^{fMet}$ in the E- (pink) and P-site (lemon), a nonstop mRNA (magenta), ArfA (density) and RF2 (dark red) in the A-site. Zoomed view (right panel) shows the conformation of important residues in decoding center when a nonstop mRNA binds.

(d) The density map coloured by local resolution in surface and slice views.

(e) Representative maps showing the refined structures of 23S rRNA (cyan), P-tRNA (lemon) and ribosomal protein (teal).

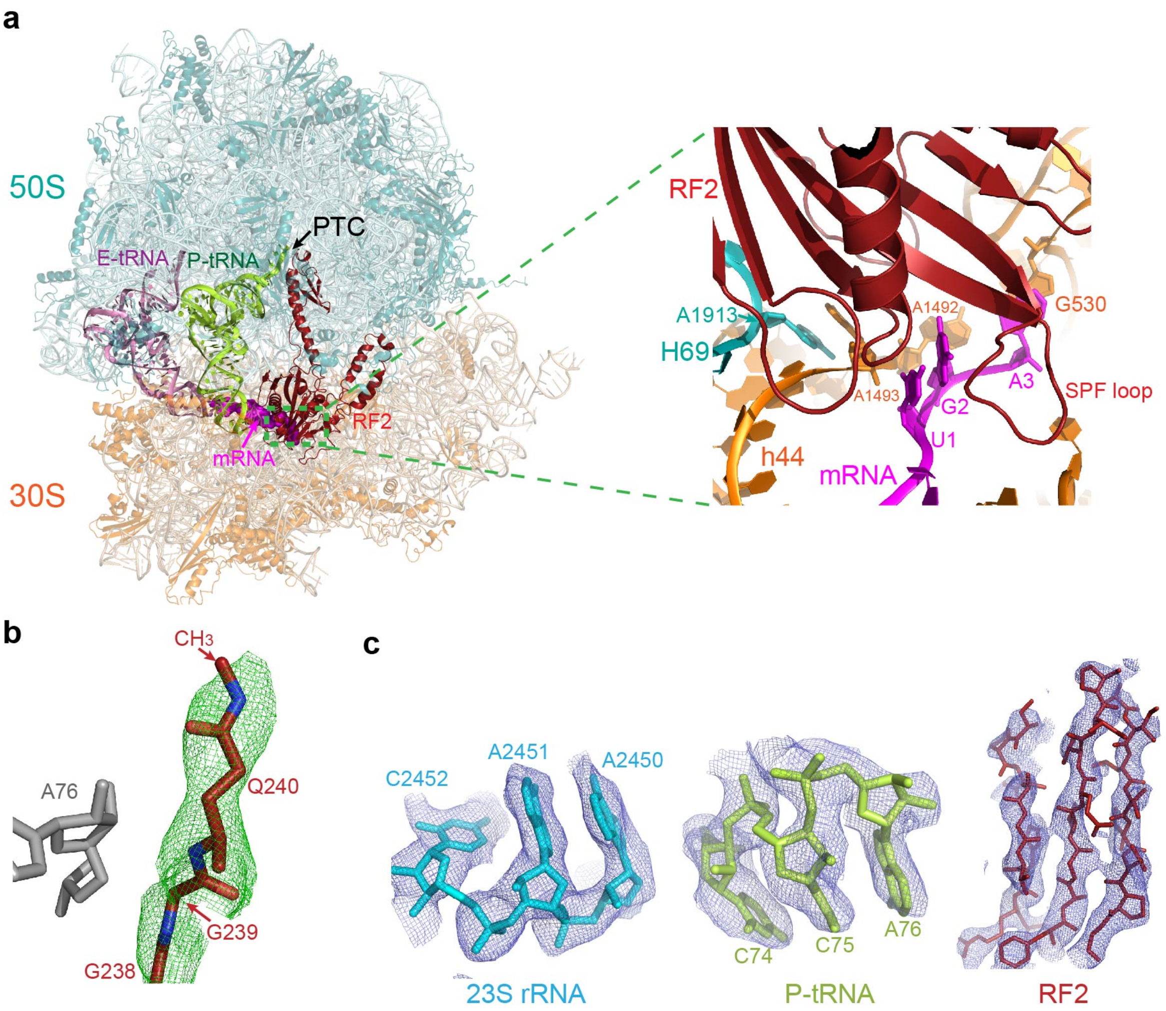

Supplementary Fig. S3 | Quality of map and structure determined by X-ray crystallography.
(a) Overall view of the canonical termination complex shows the structure of the *T. thermophilus* 70S ribosome with E-tRNA (pink), P-tRNA (lemon), mRNA (magenta) and RF2 (dark red) bound on the stop codon UGA. Zoomed view shows the important residues in the decoding center of the ribosome.
(b) σ_A weighted $3m\mathrm{F}_{obs}$-$2D\mathrm{F}_{cal}$ omit map for GGQ^m (firebrick). P-tRNA is shown as sticks in grey.
(c) Representative $2m\mathrm{F}_{obs}$-$D\mathrm{F}_{cal}$ maps showing the refined structures of 23S rRNA (cyan), P-tRNA (lemon) and RF2 (firebrick).

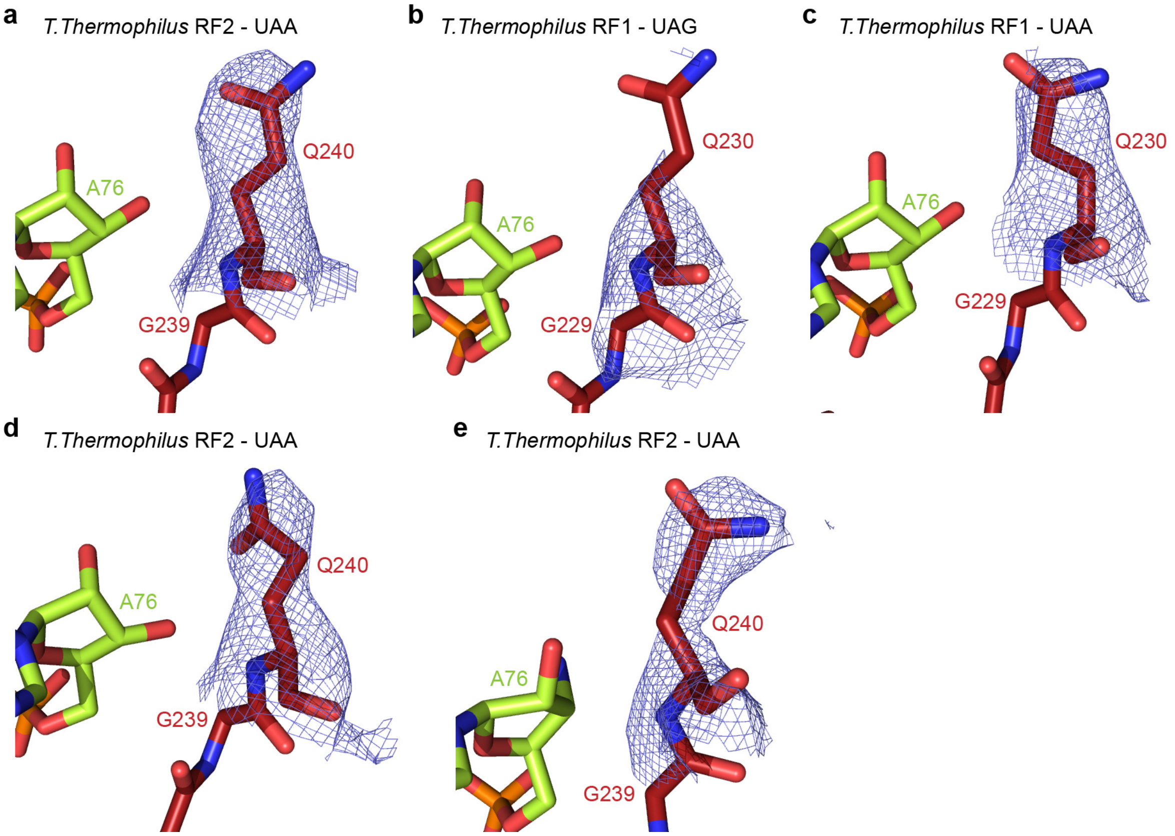

Supplementary Fig. S4 | Different comformations of the unmethylated glutamine in the PTC reported previously. Map densities of the unmethylated glutamine indicate conformational flexibility of this residue. Glutamine of the GGQ was shown in termination complexes with RF2 and stop codon UAA (**a**, PDB: 4V67) [10]; RF1 on stop codon UAG (**b**, PDB: 4V7P) [11]; RF1 and stop codon UAA (**c**, PDB: 4V63) [12]; RF2 and stop codon UGA (**d**, PDB: 4V5E) [13]; and RF2 on stop codon UAA (**e**, PDB: 4V5J) [9].

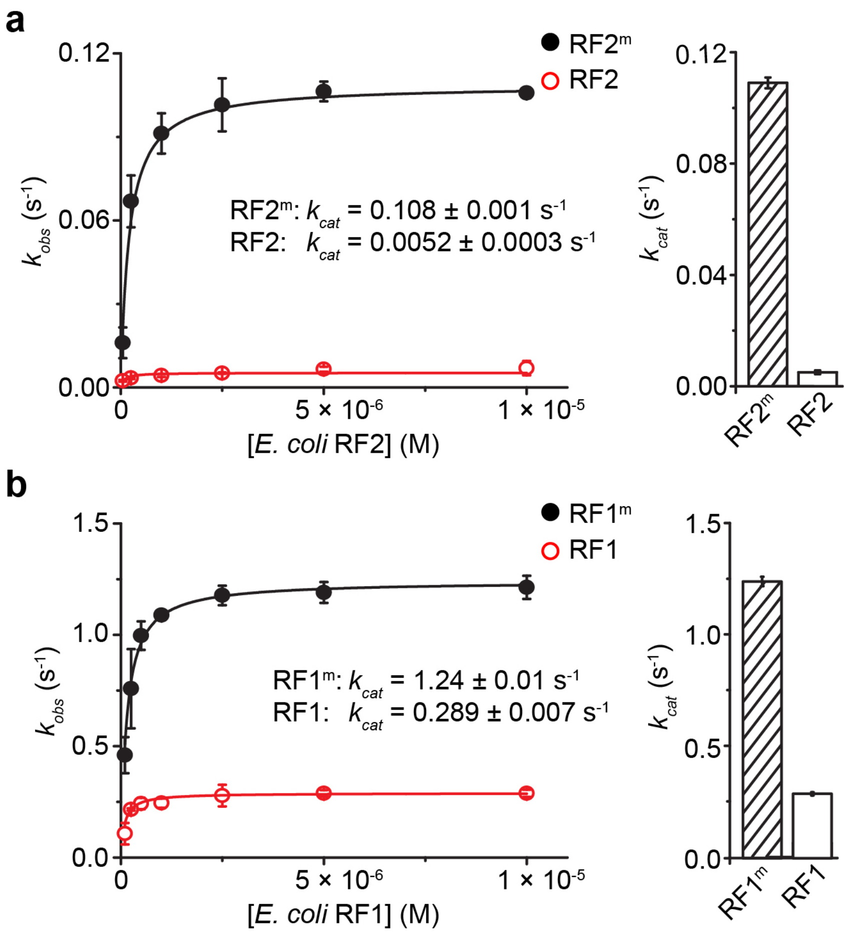

Supplementary Fig. S5 | Functional importance of the glutamine N^5 methylation.

Observed rates and rate constants k_{cat} of peptide release catalyzed by RF2 **(a)** and RF1 **(b)** in the methylated ($RF1^m$ and $RF2^m$) and un-methylated (RF1 and RF2) states on the cognate stop codon UAA in the *E. coli* ribosome.

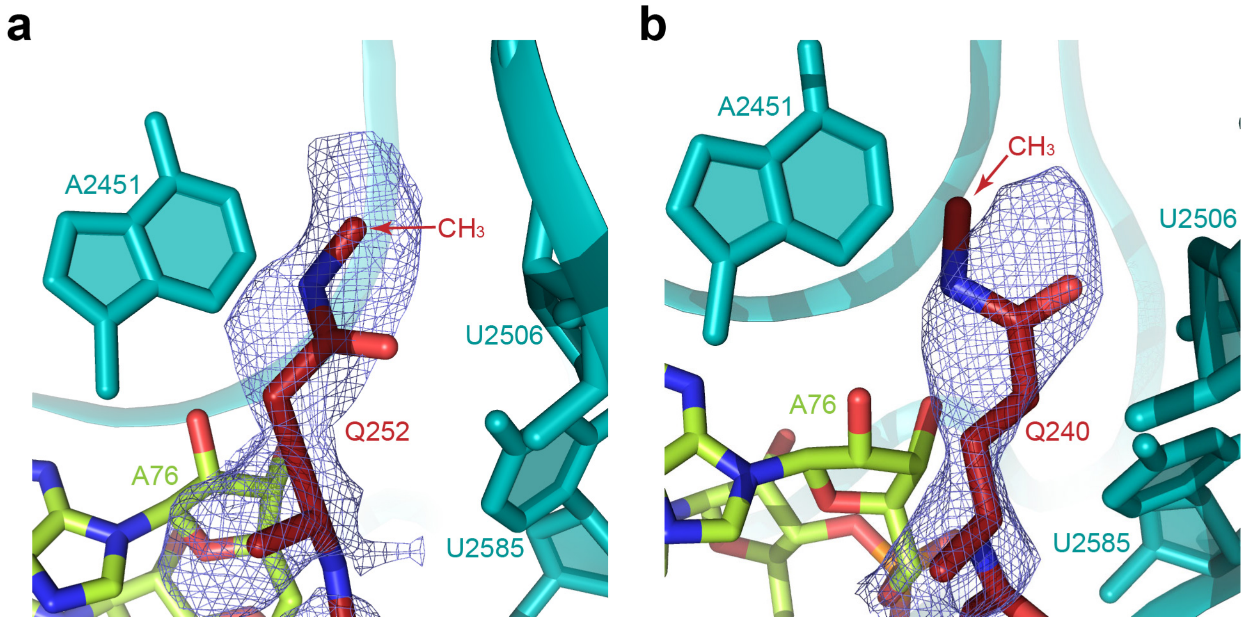

Supplementary Fig. S6 | Fitting the glutamine into the desity map with an alternative conformation.
An alternative rotamer of Gln, in which the methyl-NH group was placed next to A2451 and carbonyl group near U2506 of 23S rRNA, was fitted into the cryoEM map for the nonstop termination complex (**a**) and the X-ray map for the canonical termination complex (**b**) followed by refinement wth Refmac or Phenix. In both models, the sidechain of glutamine can not fit into the density satisfactorily. 23S rRNA, RF2 and P-tRNA are coloured in teal, firebrick and lemon.

Supplementary Table S1 | CryoEM data collection and model statistics.

	Global refinement	Local refinement over PTC
**Data Collection**		
Particles	143,372	
Pixel size (Å)	0.60	
Defocus range (µm)	-0.7 to -3.0	
Voltage (kV)	300	
Electron dose ($e^{-}Å^{-2}$)	20	
**Model composition**		
Non-hydrogen atoms	151,446	15,326
Protein residues	6,702	183
RNA bases	4,717	647
Ligands (Zn^{2+}/Mg^{2+})	2/317	0/34
**Refinement**		
Resolution (Å)	3.24	3.10
Map sharpening B-factor ($Å^2$)	-20	-50
$FSC_{average}$	0.8263	0.8736
**Rms deviation**		
Bond lengths (Å)	0.0053	0.0053
Bond angles (˚)	1.0093	0.9826
**Validation (proteins)**		
Molprobity score	2.31 (99th)	2.08 (99th)
Clashscore, all atoms	4.60 (100th)	6.86 (100th)
Good rotamers (%)	90.23	93.88
**Ramachandran plot**		
Favored (%)	95.39	94.74%
Outliers (%)	0.41	0.00
**Validation (RNA)**		
Correct sugar puckers (%)	98.49	98.92
Good backbone conformation (%)	80.87	80.22

Supplementary Table S2 | Summary of crystallographic data and refinement.

**Data collection**	
Wavelength (Å)	0.9792
Space group	$P2_12_12_1$
Cell dimensions	
a, *b*, *c* (Å)	*a* = 211.023, *b* = 452.527, *c* = 623.443
α, *β*, *γ* (˚)	*α* = *β* = *γ* = 90
Resolution (Å)	50.0 – 3.2 (3.25-3.2)
R_{means} (%)	43.9 (311.1)
$I/\sigma I$	10.29 (1.04)
Completeness (%)	100.0 (100.0)
Redundancy	41.9 (29.8)
CC1/2 (%)	99.9 (47.9)
**Refinement**	
Resolution (Å)	49.0-3.2
No. unique reflections	970,343
R_{work}/R_{free} (%)	22.29/24.74
No. non-hydrogen atoms	
Macromolecules	308,960
Ligands	480
Solvent	19
B-factors ($Å^2$)	
Macromolecules	128.73
Ligands	75.04
Solvent	64.49
R.m.s deviations	
Bond length (Å)	0.004
Bond angles (˚)	1.091
